# Gut resistome linked to sexual preference and HIV infection

**DOI:** 10.1186/s12866-024-03335-z

**Published:** 2024-06-08

**Authors:** Elisa Rubio Garcia, Maria Casadellà, Mariona Parera, Jordi Vila, Roger Paredes, Marc Noguera-Julian

**Affiliations:** 1 Department of Microbiology, CDB, Hospital Clinic, University of Barcelona, Barcelona, Spain; 2 Molecuar Core Facilty, Hospital Clínic de Barcelona, Barcelona, Spain; 3 ISGlobal Barcelona Institute for Global Health, Barcelona, Spain; 4 IrsiCaixa, Ctra de Canyet S/N, 08916 Badalona, Spain; 5 Universitat de Vic-Universitat Central de Catalunya, Vic, Spain; 6 Universitat Autònoma de Barcelona, Cerdanyola del Vallès, Spain; 7 Department of Infectious Diseasest &, Lluita Contra La SIDA Foundation, Hospital Universitari Germans Trias I Pujol, Badalona, Spain; 8 Infectious Disease Networking Biomedical Research Center (CIBERINFEC), Carlos III Health Institute, Madrid, Spain

**Keywords:** Gut resistome, HIV infection, Shotgun metagenomics, Antimicrobial resistance, Gut microbiome

## Abstract

**Background:**

People living with HIV (PLWH) are at increased risk of acquisition of multidrug resistant organisms due to higher rates of predisposing factors. The gut microbiome is the main reservoir of the collection of antimicrobial resistance determinants known as the gut resistome. In PLWH, changes in gut microbiome have been linked to immune activation and HIV-1 associated complications. Specifically, gut dysbiosis defined by low microbial gene richness has been linked to low Nadir CD4 + T-cell counts. Additionally, sexual preference has been shown to strongly influence gut microbiome composition in PLWH resulting in different *Prevotella* or *Bacteroides* enriched enterotypes, in MSM (men-who-have–sex-with-men) or no-MSM, respectively. To date, little is known about gut resistome composition in PLWH due to the scarcity of studies using shotgun metagenomics. The present study aimed to detect associations between different microbiome features linked to HIV-1 infection and gut resistome composition.

**Results:**

Using shotgun metagenomics we characterized the gut resistome composition of 129 HIV-1 infected subjects showing different HIV clinical profiles and 27 HIV-1 negative controls from a cross-sectional observational study conducted in Barcelona, Spain. Most no-MSM showed a *Bacteroides-*enriched enterotype and low microbial gene richness microbiomes. We did not identify differences in resistome diversity and composition according to HIV-1 infection or immune status. However, gut resistome was more diverse in MSM group, *Prevotella*-enriched enterotype and gut micorbiomes with high microbial gene richness compared to no-MSM group, *Bacteroides-*enriched enterotype and gut microbiomes with low microbial gene richness. Additionally, gut resistome beta-diversity was different according to the defined groups and we identified a set of differentially abundant antimicrobial resistance determinants based on the established categories.

**Conclusions:**

Our findings reveal a significant correlation between gut resistome composition and various host variables commonly associated with gut microbiome, including microbiome enterotype, microbial gene richness, and sexual preference. These host variables have been previously linked to immune activation and lower Nadir CD4 + T-Cell counts, which are prognostic factors of HIV-related comorbidities. This study provides new insights into the relationship between antibiotic resistance and clinical characteristics of PLWH.

**Supplementary Information:**

The online version contains supplementary material available at 10.1186/s12866-024-03335-z.

## Background

The increase in incidence and dissemination of multidrug-resistant organisms (MDRO) is a serious public health problem due to the high morbidity and mortality of infections caused by these microorganisms and the limited therapeutic options available for their treatment [1]. Reported risk factors for MDRO acquisition are antibiotic consumption, previous exposure to the health care system and immunosuppression. Overall, health-compromised individuals face higher risks of acquiring MDRO and experiencing worse outcomes [2–4].

People living with HIV (PLWH) are more probably colonized and/or infected by MDRO [4–8]. This population has increased rates of comorbidities, more frequent hospital admissions and receive more antibiotic treatment courses and prophylaxis compared to the general population [9, 10]. Additionally, HIV infection is associated with immunosuppression and changes in gut microbiome composition, all predisposing factors for MDRO acquisition [11, 12].

The main reservoir of MDRO and antibiotic resistance genes in humans is the gastrointestinal tract, and intestinal colonization by MDRO frequently precedes infection by these microorganisms [13, 14]. The human gut contains a highly concentrated and thriving ecosystem of microorganisms known as the intestinal microbiome. The set of antimicrobial resistance determinants (AMRD) within the gut microbiome is known as the gut resistome. The highly concentrated and diverse gut microbiome and its exposure to antibiotics and other external factors offers ample opportunities for the selection and dissemination through horizontal gene transfer of AMRD [15].

In PLWH, changes in gut microbiome have been linked to chronic immune activation. This connection is believed to potentially contribute to higher mortality rates and an increased susceptibility to clinical comorbidities associated with inflammation [16]. These imbalances in gut microbiome are characterized by a decrease in alpha-diversity, but a consistent pattern of HIV-associated microbiome composition has not been identified [11, 17]. A previous study has identified an association between gut dysbiosis, defined by low microbial gene richness, and low nadir CD4 + T-cell counts [18]. Conversely, sexual behaviour is associated with large structural changes in gut microbiome composition that result in different *Prevotella* spp. or *Bacteroides* spp. enriched enterotypes in men who have sex with men (MSM) compared to heterosexuals respectively, independently of HIV serostatus [19].

Although gut microbiome in HIV infection has been widely described, to date little is known about gut resistome composition and HIV. While most microbiome studies in PLWH have been based on 16S rRNA sequencing approach, whole metagenomic sequencing is required for gut resistome analysis, explaining the lack of information available regarding this matter. Guillén *et al* [18]. identified an enrichment of AMRD in HIV-1 infected subjects with low microbial gene richness and Bai et al [20]. reported a set of antimicrobial AMRD present only in HIV-1 subjects compared to negative controls and an enrichment of AMRD associated with tetracycline antibiotic resistance and antibiotic efflux pumps in HIV-1 subjects.

In this study we used data generated from whole metagenome shotgun sequencing to characterize the gut resistome diversity and composition in HIV-1 infection and its associations with gut microbiome composition, gut microbial gene richness, sexual preference, and other clinical factors.

## Results

### Study population

This study included 156 subjects (Table 1) comprising 129 (82.7%) HIV-1 infected patients with different clinical profiles and 27 (17.3%) negative controls recruited in Barcelona, Spain, between January and December 2014. HIV-1 infected subjects were enrolled from two tertiary HIV-1 clinics and negative controls were recruited from a cohort of HIV-negative MSM at risk of becoming infected by HIV-1 attending a community centre and HIV-1-negative partners from HIV-1-infected subjects attending the HIV clinics [19, 21, 22]. Mean age of included patients was 43 years, most were male (79%) and of Caucasian ethnicity (79%). All included patients were classified according to sexual preference in MSM (*n* = 100) and no-MSM (*n* = 56), according to faecal microbiome cluster in *Bacteroides* (*n* = 63) or *Prevotella* (*n* = 93) enriched enterotypes [19] and according to microbial gene richness values obtained by whole faecal metagenome shotgun sequencing in high-gene count (HGC) (*n* = 53) or low-gene count (LGC) (*n* = 103) [18]. Low microbial gene counts have been previously linked to gut dysbiosis in different gut inflammatory diseases [23]. Additionally, in a previous study conducted in the same cohort of patients a significant and independent dose–effect association between nadir CD4 + T-cell counts and LGC was identified [18]. Most MSM showed a *Prevotella* enriched enterotype (88%) and no-MSM a *Bacteroides* enriched enterotype (91%) as previously described [19]. Regarding gene richness, most no-MSM subjects presented gut microbiome with LGC (88%). Subjects in the no-MSM group were older and showed lower Nadir CD4 + T-cell counts compared to MSMs.

**Table 1:** Patient's characteristics according to sexual preference and gene richness

	**Overall**, *N* = 156	**Sexual preference**			**Gene Richness**		
		**MSM**, *N* = 100	**no MSM**, *N* = 56	***p*-value**^*2*^	**HGC**, *N* = 53	**LGC**, *N* = 103	***p*-value**^*1*^
**Age, median (IQR)**	43 (35–51)	38 (34–46)	50 (42–54)	** < 0.001**	38 (35–46)	46 (36–53)	**0.024**
**Gender**				** < 0.001**			**0.018**
Women, n (%)	31 (20)	0 (0)	31 (55)		5 (9.4)	26 (25)	
Men, n (%)	124 (79)	99 (99)	25 (45)		47 (89)	77 (75)	
Transgender women, n (%)	1 (0.6)	1 (1.0)	0 (0)		1 (1.9)	0 (0)	
**Ethnicity**				0.2			**0.005**
Asiatic, n (%)	1 (0.6)	0 (0)	1 (1.8)		1 (1.9)	0 (0)	
Caucasian, n (%)	124 (79)	78 (78)	46 (82)		36 (68)	88 (85)	
Hispanic-Latin, n (%)	28 (18)	21 (21)	7 (13)		16 (30)	12 (12)	
Other, n (%)	3 (1.9)	1 (1.0)	2 (3.6)		0 (0)	3 (2.9)	
**BMI,** median (IQR)	23.8 (22.0–26.1)	24.3 (22.3–26.2)	23.5 (20.9–25.2)	0.053	24.4 (22.3–26.3)	23.7 (21.8–25.5)	0.2
Missing values	18	16	2		7	11	
**HIV-1 status**				**0.012**			**0.002**
Negative, n (%)	27 (17)	23 (23)	4 (7.1)		16 (30)	11 (11)	
Positive, n (%)	129 (83)	77 (77)	52 (93)		37 (70)	92 (89)	
**HIV-1 phenotype**							
Concordant, n (%)	53 (34)	28 (28)	25 (45)		11 (21)	42 (41)	
Discordant, n (%)	18 (12)	6 (6.0)	12 (21)		3 (5.7)	15 (15)	
Early-treated, n (%)	13 (8.3)	12 (12)	1 (1.8)		5 (9.4)	8 (7.8)	
Elite controller, n (%)	8 (5.1)	3 (3.0)	5 (8.9)		3 (5.7)	5 (4.9)	
Late presenter, n (%)	11 (7.1)	8 (8.0)	3 (5.4)		2 (3.8)	9 (8.7)	
ART-naive, n (%)	15 (9.6)	13 (13)	2 (3.6)		7 (13)	8 (7.8)	
Viremic controller, n (%)	11 (7.1)	7 (7.0)	4 (7.1)		6 (11)	5 (4.9)	
HIV-1 negative, n (%)	27 (17)	23 (23)	4 (7.1)		16 (30)	11 (11)	
**Antiretroviral treatment**, n (%)	66 (42)	40 (40)	26 (46)	0.4	16 (30)	50 (49)	**0.028**
**Gene richness**				** < 0.001**			
HGC, n (%)	53 (34)	46 (46)	7 (13)				
LGC, n (%)	103 (66)	54 (54)	49 (88)				
**Sexual preference**							** < 0.001**
MSM, n (%)	100 (64)				46 (87)	54 (52)	
no MSM, n (%)	56 (36)				7 (13)	49 (48)	
**Microbiome cluster**				** < 0.001**			** < 0.001**
*Bacteroides*, n (%)	63 (40)	12 (12)	51 (91)		7 (13)	56 (54)	
*Prevotella*, n (%)	93 (60)	88 (88)	5 (8.9)		46 (87)	47 (46)	
**Antibiotic intake, previous 3 months,** n (%)	2 (1.3)	2 (2.0)	0 (0)	0.5	0 (0)	2 (1.9)	0.5
**Antibiotic intake, previous 6 months,** n (%)	35 (22)	20 (20)	15 (27)	0.3	9 (17)	26 (25)	0.2
**HIV-1 RNA, copies/mL**^**2**^				**0.044**			**0.034**
Undetectable, n (%)	85 (66)	45 (58)	40 (78)		19 (51)	66 (73)	
< = 10.000, n (%)	22 (17)	15 (19)	7 (14)		11 (30)	11 (12)	
> 10.000, n (%)	21 (16)	17 (22)	4 (7.8)		7 (19)	14 (15)	
Missing values	1	0	1		0	1	
**CD4 + T-cell counts**^**3**^, median (IQR)	705 (469–856)	727 (490–851)	636 (288–934)	0.8	772 (570–860)	644 (289–853)	0.11
Missing values	1	1	0		0	1	
**Nadir CD4 + T-cell counts**^**3**^, median (IQR)	337 (140–529)	372 (209–577)	244 (91–438)	**0.005**	443 (339–601)	280 (113–492)	**0.002**
Missing values	2	2	0		1	1	
**CD8 + T-cell counts**^**3**^, median (IQR)	777 (576–1,012)	779 (627–983)	777 (478–1,027)	0.6	749 (604–1,158)	792 (559–991)	0.3
Missing values	1	1	0		1	0	
**CD4 + /CD8 + ratio**^**3**^, median (IQR)	0.84 (0.52–1.22)	0.83 (0.55–1.19)	0.92 (0.46–1.34)	0.7	0.81 (0.55–1.16)	0.88 (0.49–1.32)	> 0.9
Missing values	2	2	0		1	1	

*ART* antiretroviral treatment, *BMI* body mass index, *HGC* high-gene count, *IQR* interquartile range, *LGC* low-gene count, *MSM* men who have sex with men

^1^Wilcoxon rank sum test; Fisher’s exact test; Pearson’s Chi-squared test

^2^^,3^Values obtained only for HIV-1 positive subjects (*n* = 129)

### Gut resistome diversity

A total of 308 different AMRD grouped in 97 antimicrobial resistant (AMR) gene families were identified in the overall analysed samples. The most abundant AMR gene families in this study were tetracycline-resistant ribosomal protection protein, CfxA beta-lactamase, 23S rRNA with mutation conferring resistance to macrolide antibiotics, 16S rRNA with mutation conferring resistance to aminoglycoside antibiotics and Erm 23S ribosomal RNA methyltransferase conferring resistance to macrolide, lincosamide and streptogramin (MLS) antibiotics.

**Fig. 1:**
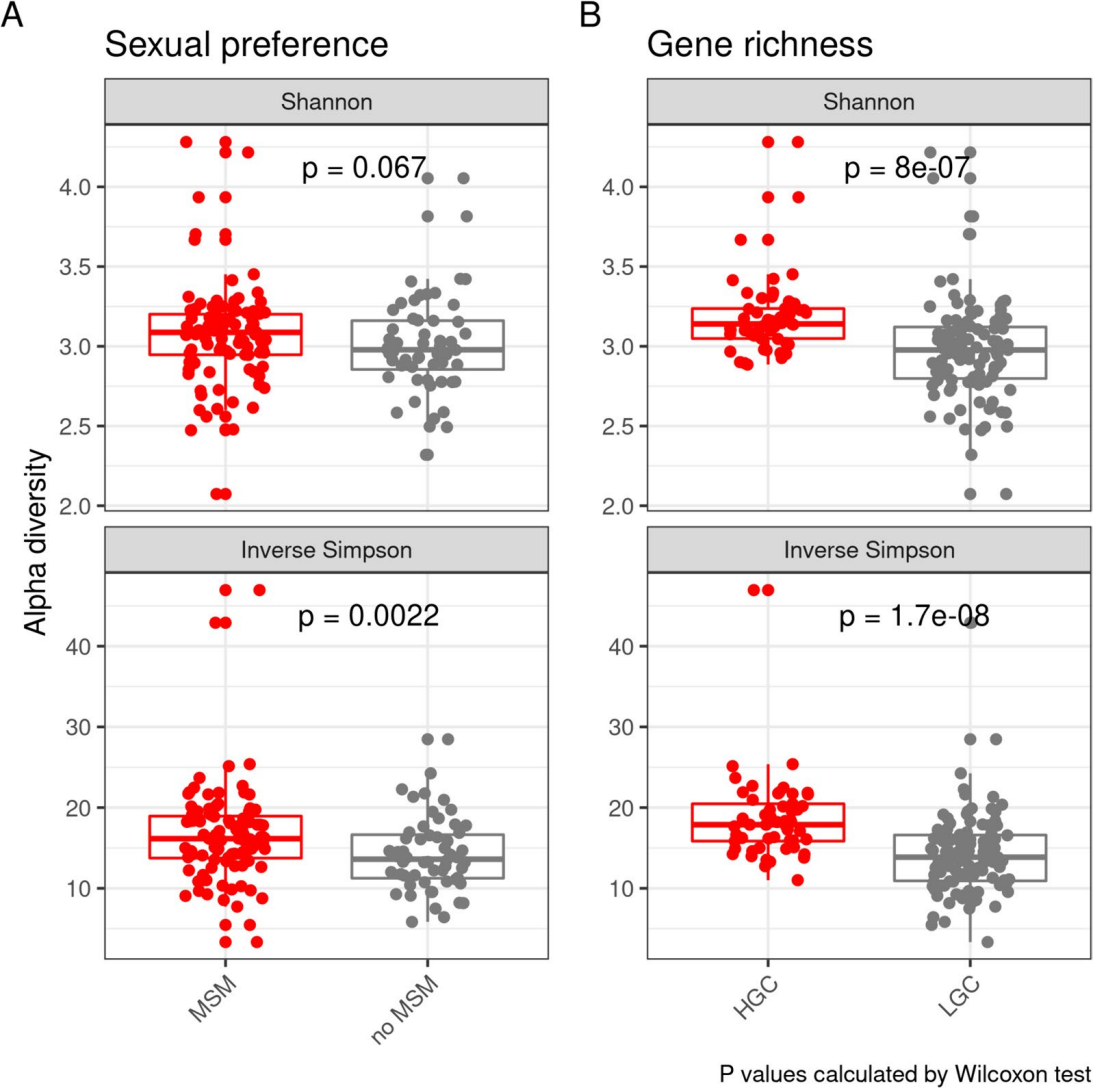
Differences in gut resistome alpha diversity measured by Shannon and Inverse Simpson diversity indexes according to sexual preference (**A**) and gene richness (**B**). Group differences were calculated using one-sided Wilcoxon tests

No differences in gut resistome alpha diversity and composition were identified according to HIV-1 infection status, HIV-1 phenotype or whether subjects had initiated antiretroviral treatment or had previously taken antibiotics at the time of inclusion. Additionally, we did not identify significant correlations between gut resistome alpha diversity and CD4 + T-cell counts, nadir CD4 + T-cell counts, CD8 + T-cell counts and CD4 + /CD8 + ratio. However, we identified a significantly more diverse and a tendency towards a richer gut resistome in MSM compared to no-MSM subjects (Fig. 1A). The same differences were observed when comparing *Prevotella* and *Bacteroides* enriched enterotypes (sFigure 1). Regarding gene richness, a significantly higher alpha resistome diversity was identified in HGC compared to LGC microbiomes (Fig. 1B).

**Fig. 2:**
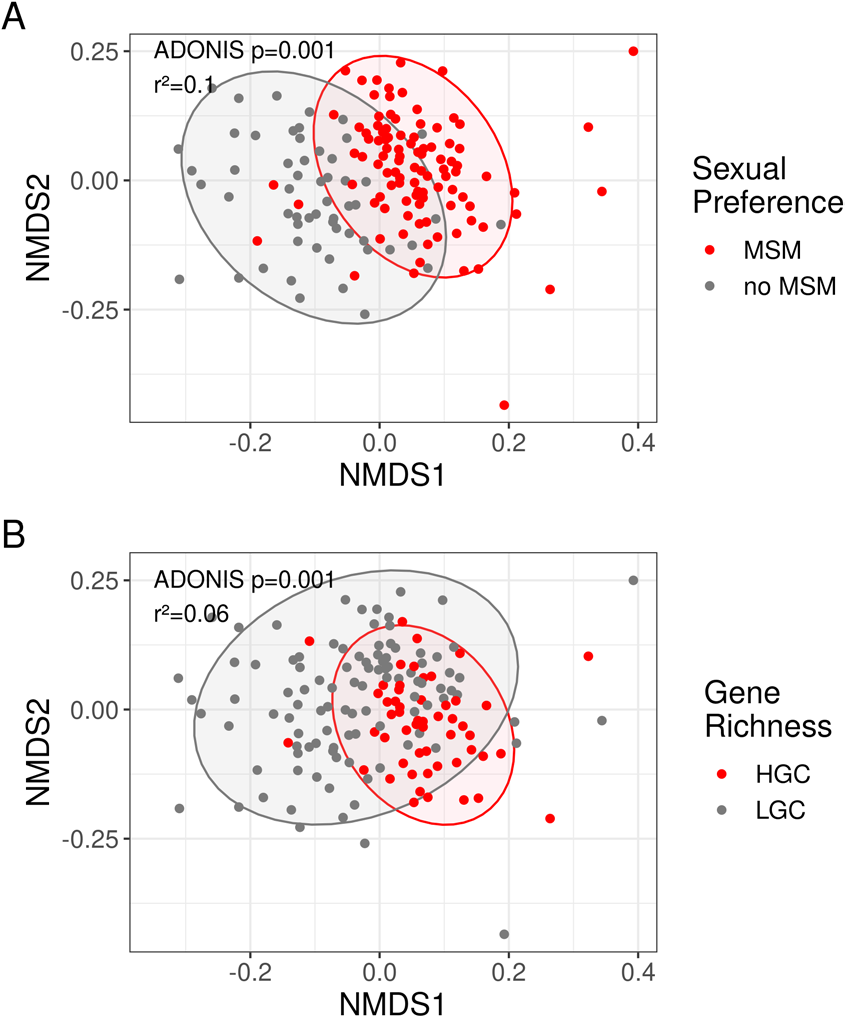
Non-metric multidimensional scaling (NMDS) plot based on resistome Bray–Curtis (BC) dissimilarity between samples stratified per sexual preference (**A**) and gene richness (**B**). Ellipses represent 95% confidence intervals. The stress of the ordination effect sizes (r^2^) calculated by PERMANOVA tests and corresponding *p*-values are shown in the plots

When analysing gut resistome composition, beta-diversity analyses showed significantly different resistome composition according to sexual preference (R^2^ = 0.1, *p*-value = 0.001), microbiome cluster (R^2^ = 0.1, *p*-value = 0.001) and gene richness (R^2^ = 0.06, *p*-value = 0.001) (Fig. 2, sFigure2). Of note, sequencing depth did not influence significantly gut resistome composition, validating the normalization method used in this study (RPKM). Initially, a univariate PERMANOVA analysis was conducted identifying a set of significant variables which were included in the sequential and marginal multivariate PERMANOVA analyses. The multivariate analysis showed that sexual preference, microbiome cluster, HIV-1 phenotype and microbiome gene richness remained independently significant contributing to differences in gut resistome composition. (Table 2, sTable 2). Of note, in the marginal multivariate analysis a decrease in R^2^ value was observed for microbiome cluster and sexual preference, demonstrating the high correlation between both variables. When pairwise comparisons were performed according to the different HIV-1 phenotypes, only Discordant *versus* ART-naïve and HIV-1 negative *versus* Elite controller comparisons remained significant (sTable 3).

**Table 2:** Univariate and sequential multivariate PERMANOVA analysis

	**Univariate analysis**		**Multivariate analysis**		**Multivariate analysis (missing values)**	
	***p*-value**	**R**^**2**^** value**	***p*-value**	**R**^**2**^** value**	***p*-value**	**R**^**2**^** value**
**Sexual preference**	**0.001**	0.1	**0.001**	0.1	**0.001**	0.1
**Microbiome cluster**	**0.001**	0.1	**0.001**	0.02	**0.001**	0.02
**HIV-1 phenotype**	**0.001**	0.08	**0.011**	0.05	**0.011**	0.06
**Gene richness**	**0.001**	0.06	**0.001**	0.05	**0.001**	0.04
**Gender**	**0.001**	0.05	0.692	0.01	0.47	0.01
**Age**	**0.001**	0.03	0.127	0.01	0.107	0.01
**Nadir CD4 + T-cell counts**	**0.001**	0.02	-	-	0.986	0
**HIV-1 RNA, copies/mL**	0.03	0.03	-	-	-	-
**Ethnicity**	0.048	0.02	-	-	-	-
**Antibiotic intake, previous 3 months**	0.062	0.01	-	-	-	-
**Antibiotic intake, previous 6 months**	0.167	0.01	-	-	-	-
**Sequencing depth**	0.206	0.01				
**BMI**	0.234	0.01	-	-	-	-
**HIV-1 status**	0.273	0.01	-	-	-	-
**CD8 + T-cell counts**	0.446	0.01	-	-	-	-
**Antiretroviral treatment**	0.529	0.01				
**CD4 + T-cell counts**	0.613	0.01	-	-	-	-
**CD4 + /CD8 + ratio**	0.683	0.01	-	-	-	-

*BMI* Body mass index

### Differentially abundant antibiotic resistance determinants

We evaluated differentially abundant AMRD according to sexual preference, microbiome gene richness (Table 3) and microbiome cluster (sTable 4) identifying a set of significantly enriched determinants. Additionally, to control for gender, we assessed differentially abundant AMRD according to sexual preference in a subset of samples that excluded women, as none belonged to the MSM group (sTable 5).

**Table 3:** Differentially abundant antimicrobial resistance gene families according to Sexual preference and Gene richness

Antimicrobial resistance gene family	Sexual preference						Gene Richness						Drug classes
	**MSM**^a^	**no MSM**^a^	***p*-value**	**adjusted *p*-value**	**log2FC**	**Group**^b^	**HGC**^a^	**LGC**^a^	***p*-value**	**adjusted *p*-value**	**log2FC**	**Group**^b^	
CblA beta-lactamase	0	8111	4.56E-19	2.10E-17	-3.58	no MSM	0	2333	1.32E-06	3.18E-05	-2.55	LGC	Cephalosporin
sulfonamide resistant sul	0	350	7.32E-08	6.73E-07	-3.37	no MSM	-	-	-	-	-	-	Sulfonamide
ABC-F ATP-binding cassette ribosomal protection protein	1298	9316	6.41E-15	9.82E-14	-2.31	no MSM	1209	4203	5.68E-05	6.54E-04	-1.63	LGC	Lincosamide, Macrolide, Oxazolidinone, Phenicol, Pleuromutilin, Streptogramin, Tetracycline
Major facilitator superfamily (MFS) antibiotic efflux pump	4662	23300	2.08E-14	2.39E-13	-1.94	no MSM	-	-	-	-	-	-	Macrolide, Tetracycline, Fluoroquinolone, Nucleoside, Aminoglycoside, Cephalosporin, Peptide, Rifamycin, Carbapenem, Penam, Fosfomycin, Lincosamide, Phenicol, acridine dye, disinfecting agents and intercalating dyes, Benzalkonium, Chloride, Rhodamine
lincosamide nucleotidyltransferase (LNU)	1767	4876	4.25E-04	1.63E-03	-1.21	no MSM	-	-	-	-	-	-	Lincosamide
23S rRNA with mutation conferring resistance to streptogramins antibiotics	23,209	28,260	7.54E-03	2.17E-02	-0.22	no MSM	-	-	-	-	-	-	Streptogramin
tetracycline-resistant ribosomal protection protein	101,674	119,180	4.17E-03	1.28E-02	-0.15	no MSM	-	-	-	-	-	-	Tetracycline
23S rRNA with mutation conferring resistance to macrolide antibiotics	81,659	66,531	1.47E-06	9.64E-06	0.32	MSM	88,954	71,266	3.55E-05	5.45E-04	0.30	HGC	Macrolide
ANT(6)	8626	3207	4.17E-03	1.28E-02	0.34	MSM						-	Aminoglycoside
16 s rRNA with mutation conferring resistance to aminoglycoside antibiotics	64,624	49,901	5.63E-04	1.99E-03	0.37	MSM						-	Aminoglycoside
23S rRNA with mutation conferring resistance to pleuromutilin antibiotics	15,126	12,043	1.30E-06	9.64E-06	0.39	MSM						-	Pleuromutilin
CfxA beta-lactamase	89,898	56,298	2.96E-04	1.24E-03	0.55	MSM						-	Cephamycin
ACI beta-lactamase	1920	0	1.40E-16	3.22E-15	3.28	MSM	1819	625	5.28E-03	3.47E-02	0.12	HGC	Cephalosporin, Penam
APH(3')	-	-		-	-	-	4206	2192	1.11E-03	8.52E-03	-0.10	LGC	Aminoglycoside
16S rRNA with mutation conferring resistance to tetracycline derivatives	-	-		-	-	-	3451	499	6.44E-03	3.70E-02	0.99	HGC	Tetracycline
chloramphenicol acetyltransferase (CAT)	-	-		-	-	-	2939	406	1.38E-06	3.18E-05	1.14	HGC	Phenicol

*HGC* high-gene count, *LGC* low-gene count, *MSM* men who have sex with men

^a^median RPKM (Reads Per Kilobase per Million mapped reads)

^b^Group where the antimicrobial resistance gene family is enriched

We identified that MSM microbiome were enriched in 16S rRNA with mutations conferring resistance to aminoglycoside antibiotics, 23S rRNA with mutations conferring resistance to macrolide and pleuromutilin antibiotics, ANT (6), enzyme conferring resistance to aminoglycosides and *CfxA* and *ACl* beta-lactamases conferring resistance to cephalosporin and cephamycin antibiotics, respectively. On the other hand, MSM were depleted in *CblA* beta-lactamase conferring resistance to cephalosporins, sulfonamide resistant *sul*, ABC-F ATP-binding cassette ribosomal protection protein and major facilitator superfamily (MFS) antibiotic efflux pump conferring resistance to different antibiotic classes, 23S rRNA with mutation conferring resistance to streptogramins antibiotics,tetracycline-resistant ribosomal protection protein (Table 3). Most AMRD enriched in MSM and no-MSM groups were also significantly enriched in *Prevotella* and *Bacteroides* enterotypes, respectively (sTable 4). Additionally, the most significantly enriched AMRD according to sexual preference identified in all patients, were also identified in the subset of samples excluding women (sTable 5).

According to gene richness *CblA* beta-lactamases ABC-F ATP-binding cassette ribosomal protection protein and APH (3') resistance determinants were enriched in LGC group. Resistance determinants enriched in HGC group were 23S rRNA with mutation conferring resistance to macrolide antibiotics, 16S rRNA with mutation conferring resistance to tetracycline derivatives, *ACI* beta-lactamase and chloramphenicol acetyltransferase (CAT).

**Fig. 3:**
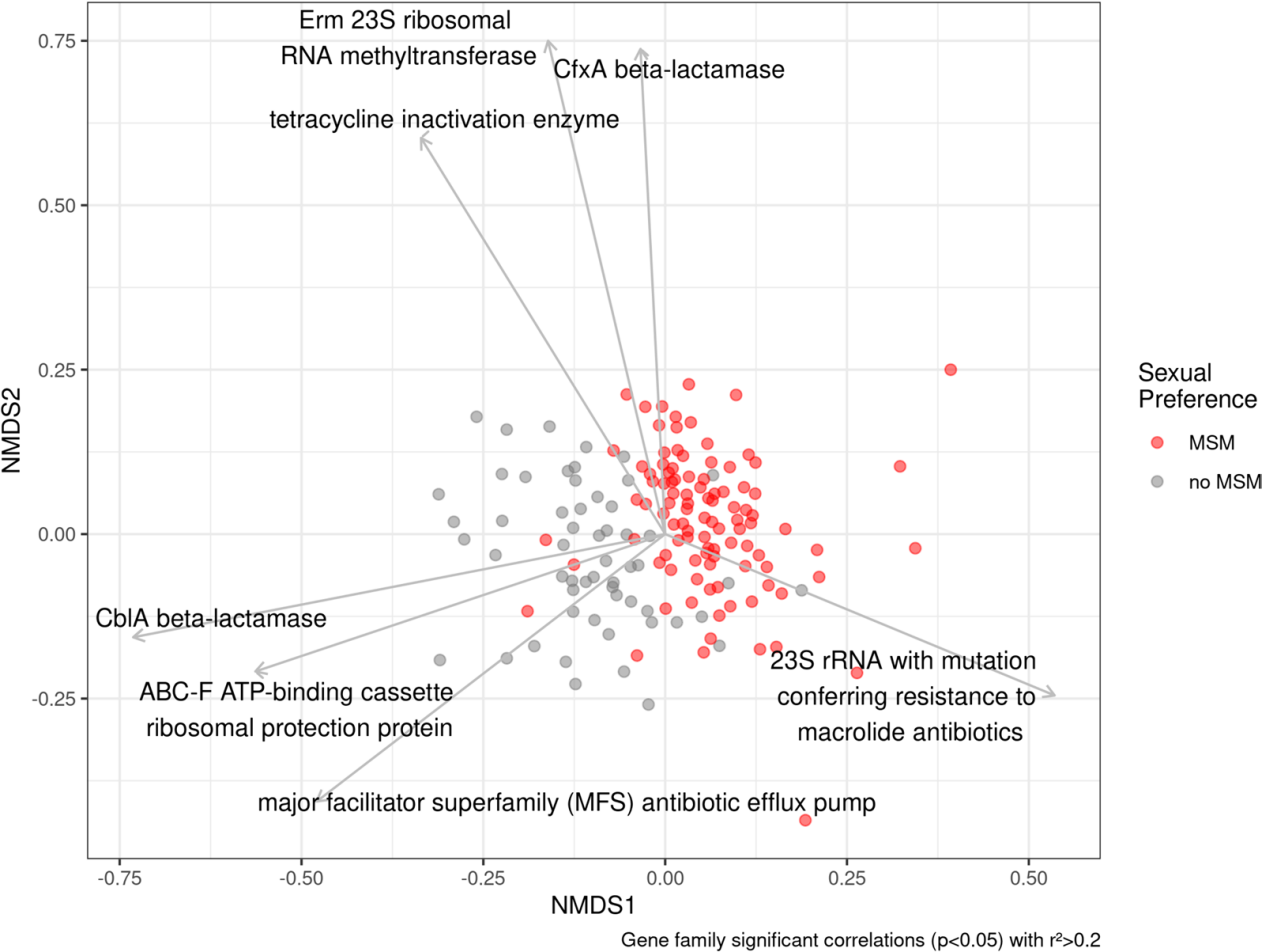
Non-metric multidimensional scaling (NMDS) plot based on resistome Bray–Curtis (BC) dissimilarity between samples. Dot colours represent sexual preference. Antimicrobial resistance gene families significantly (*p* < 0.05) associated to an environmental vector of more than 0.2 NMDS length are represented

Not surprisingly, a set of differentially abundant AMRD were among determinants with higher loadings on ordination components in the resistome beta-diversity composition (Fig. 3, sFigure 3).

Namely, *CblA* beta-lactamase, ABC-F ATP-binding cassette ribosomal protection protein (both AMRD enriched in no-MSM, *Bacteroides* and LGC groups) and MSF antibiotic efflux pump (enriched in no-MSM and *Bacteroides* groups) showed negative significant loading towards NMDS component 1. On the other hand, 23S rRNA with mutation conferring resistance to macrolide antibiotics (enriched in MSM, *Prevotella* and HGC groups) showed a positive significant loading towards NMDS component 1. Finally, *CfxA* beta-lactamase (enriched in MSM and*Prevotella* groups) showed a positive significant loading towards NMDS component 2. As expected, the loadings showed a correlation with the grouping variables in which the different AMRD were enriched (Fig. 3, sFigure 3).

We did not identify a clear tendency towards antibiotic classes the identified AMRD were conferring resistance to and sexual preference, gene richness or microbiome cluster.

## Discussion

The present study aimed to detect associations between different microbiome features linked to HIV-1 infection and gut resistome composition. By analysing the AMRD within the gut microbiome, we sought to gain insight into the potential impact of HIV-1 infection on gut microbial community at the resistome level and the relationship with the increased incidence of MDRO colonization and infection in HIV population.

We identified differences in gut resistome diversity and composition according to sexual preference, gut microbiome enterotype and gut microbiome gene richness, but not related to HIV-1 infection or immune status. Gut resistome was more diverse in MSM group and *Prevotella* enriched, HGC gut microbiomes compared to no-MSM group, and *Bacteroides* enriched and LGC gut microbiomes. Additionally, we identified a set of differentially abundant AMRD according to the defined groups. To our knowledge, this is the first study to exhaustively characterize gut resistome composition in HIV-1 infection.

It has been shown that sexual preference has a profound impact on gut microbiome composition, and it might have been a confounding factor for HIV-related microbiome studies [17]. These changes in gut microbiome composition according to sexual preference could also reflect on gut resistome composition. Gut microbiome of MSM has been characterized by a higher abundance of *Prevotella* spp. and a depletion of *Bacteroides* spp. compared to no-MSM [17, 19, 24]. Unprotected receptive anal intercourse, rectal douching or enema or the use of hyperosmotic lubricants have been suggested as influencing factors for microbiome changes in MSM population [17, 25]. However, these factors are not limited to MSM and not all MSM practice them with the same frequency, thus, requiring further investigation. As for health implications, MSM associated microbiome has been shown to be related to increased immune activation and bacterial translocation [16, 26] in HIV population.

Recent studies have shown that gut microbiome of MSM is altered independently of HIV-1 infection status, showing higher levels of gastrointestinal inflammation [27] and contributing to an increased risk of HIV-1 infection [28]. On the other hand, *Prevotella* enriched enterotype in other populations has shown contradictory health effects being related to anti-inflammatory effects of diet [29] and lower risk of infection and mortality in critical care patients [30] but also to different inflammatory conditions [31, 32].

In other chronic diseases associated with significant changes in gut microbiome composition like type 2 diabetes, cirrhosis and cardiovascular diseases, higher abundance or diversity of gut AMRD have been associated with poorer outcomes [33–36]. Consistent with these observations, our study reveals higher resistome diversity in MSM subjects with *Prevotella*-enriched gut microbiomes, which in turn have been linked to immune activation in PLWH and increased HIV-associated comorbidities and mortality [10].

On the other hand, we also identified a different resistome composition and higher resistome diversity associated to HGC microbiomes. Lower microbiome gene richness has been previously linked to gut dysbiosis and higher risk of obesity-associated co-morbidities like type 2 diabetes, cardiometabolic diseases and inflammation in microbiome studies using shotgun metagenomics [23, 37–39]. Additionally, in a previous study performed on the same cohort of patients, an association between microbiome gene richness and nadir CD4 + T-cell counts was identified [18]. In turn, lower nadir CD4 + T-cell counts are related to late HIV diagnosis being a marker of immune damage, systemic inflammation, and clinical complications in PLWH [40]. Despite finding a relationship between gut resistome and microbiome gene richness, we could not detect differences in resistome composition and diversity related to nadir CD4 + T-cell counts. These results contradict previous findings associating higher resistome diversity with poorer outcomes suggesting that the intestinal resistome introduces a novel dimension of information to the diverse correlations between gut microbiome composition and various clinical variables among PLWH.

Our study showed that AMRD conferring resistance to tetracycline, beta-lactams, aminoglycoside, and MLS antibiotics were the most dominant in the human gut resistome, in agreement with previous studies [35, 36, 41].

In the differential abundance of antimicrobial resistance determinant analysis between significant variables, a clear trend of increased resistance determinants based on the antibiotic to which they confer resistance to was not identified. The resistance determinants showing higher fold change values between groups conferred resistance to cephalosporines and were enriched in both no-MSM/HGC/*Bacteroides* groups (CblA beta-lactamase) and MSM/LGC/*Prevotella* groups (ACI beta-lactamase). CblA beta-lactamase is a species-specific class A bet-lactamase found in *Bacteroides uniforms* [42] and ACI beta-lactamase has been detected to be harboured by *Acidominococcus intestini* and other *Negativicutes* in human gut metagenomes [43, 44]. Thus, bacterial composition of gut microbiome would explain the higher abundance of CblA beta-lactamase in *Bacteroides* enriched enterotypes and of ACl beta-lactamase in *Prevotella* enriched enterotype, as *Acidominoccus* genera was found to be positively correlated with *Prevotella* in the microbiome of studied subjects [19]. Additionally, co-occurrence of class A beta-lactamases and different *Bacteroides* species has been previously reported in human gut resistome studies [36, 41]. A co-occurrence between *Prevotella copri* and CfxA beta-lactamase has also been reported [36], an AMRD enriched in MSM/LGC/*Prevotella* groups in our study.

The influence of microbiome composition in shaping resistome structure has been widely described in human gut analyses [36, 41, 45] and in environmental samples [46]. In line with our results, Qiu et al [36]. analysed gut resistome in healthy individuals and subjects with various diseases identifying a higher resistome abundance in patients with cirrhosis and type 2 diabetes but AMRD differences were mostly related to specific disease-associated bacteria rather than identifying an AMRD consistent pattern.

There are several limitations to this study. Firstly, the sample size is relatively small and was conducted as a cross-sectional study, restricting the ability to establish causal relationships. Additionally, the study was not designed to include a control group of HIV-1 negative no-MSM resulting in an underrepresentation of this group and variables such as frequency of receptive anal intercourse were not collected. Another limitation is the depth of coverage and actual capabilities of resistome profiling such as the incapacity to detect AMRD expression levels. Moreover, the lack of standardized methods for resistome analysis hampers the comparability and reproducibility of results across studies [47, 48]. Lastly, the lack of phenotypic resistome profiling hampers our ability to link phenotype to genotype data and analyse the direct impact of resistome on MDRO colonization and infection in PLWH. Ongoing studies are being conducted by our group to investigate this relationship in independent cohorts in.

Our results describe a strong relationship between gut resistome composition and host variables that are frequently associated with gut microbiome, such as microbiome enterotype, microbial gene richness or sexual preference. These host variables have been found to be associated to immune activation and lower Nadir CD4 + T-Cell counts that are prognostic factors of HIV-related comorbidities.

## Conclusion

In conclusion, this study identified that sexual preference, gut microbiome enterotype and gut microbial gene richness influence gut resistome composition in PLWH. It was observed that gut resistome diversity was notably higher in individuals who identified as MSM, exhibited a *Prevotella*-enriched enterotype, and possessed a HGC microbiome. Our findings indicate that changes in the gut microbiome associated with these factors in PLWH shape gut resistome composition. This study provides new insights into the relationship between antibiotic resistance and clinical characteristics of PLWH.

## Methods

### Study design

This was a cross-sectional study conducted in Barcelona, Catalonia, Spain, involving HIV-1 infected participants with different virologic and immunologic phenotypes and HIV-negative controls. Further details about the cohort design and characteristics have been published elsewhere [18, 19]. The study was carried out between January and December 2014. HIV-1 infected subjects were recruited from the HIV Clinics of two tertiary care hospitals, Germans Trias i Pujol and Vall d'Hebrón. HIV-negative controls were primarily recruited from a prospective cohort of HIV-negative (MSM) and were at risk of HIV-1 infection [22]. These individuals attended regular medical and counselling visits, including HIV-1 testing, at a community-based centre in Barcelona [21]. Additional controls were HIV-negative partners of HIV-1-infected subjects who were attending the HIV clinics. The study included participants aged between 18 and 60 years with a BMI within the range of 18.5 and 30. Exclusion criteria were dietary deviations from a usual diet, recent antibiotic use, pregnancy or intent to become pregnant, current drug consumption or alcohol abuse, chronic digestive diseases, surgical resection of the intestines (except for appendectomy), autoimmune diseases, and symptomatic chronic liver disease or hepatic insufficiency defined as a Child–Pugh C score.

HIV-1-infected participants were classified according to their virological and immunological status into seven mutually excluding HIV-1 phenotypes: (a) elite controllers: HIV-1 RNA < 50 copies/mL during at least 2 years in the absence of ART; (b) viremic controllers: HIV-1 RNA between 50 and 2000 copies/mL during at least 2 years in the absence of ART; (c) early-treated: ART initiation during the first 6 months after HIV-1 infection, HIV-1 RNA levels < 50 copies/mL during at least the 3 last months and with no HIV-1 RNA blips after achieving HIV-1 RNA < 50 copies/mL; (d) ART-naïve: HIV-1 RNA > 10,000 copies/mL, nadir CD4 + T-cell counts > 500 cells/mm3 and no ART exposure; (e) immune concordant: HIV-1 RNA levels < 50 copies/mL, and CD4 + T-cell counts > 500 cells/mm3 during at least 2 years; (f) immune discordant: HIV-1 RNA < 50 copies/mL and CD4 + T-cell < 300 cells/mm3 during at least 2 years under ART; and (g) late presenters: CD4 + T-cell counts < 200 cells/mm3 at HIV-1 diagnosis and no ART exposure.

### Data collection

A centralized database specifically designed for this study (OpenClinica, 2015 OpenClinica, LLC) was used to gather clinical and laboratory data employing a standardized questionnaire [19]. Microbiome enterotype classification in *Prevotella* and *Bacteroides*-enriched categories and microbiome gene richness classification in HGC and LGC categories was obtained from previously reported analyses [18, 19].

### DNA extraction and sequencing

Sample processing, DNA extraction and microbial sequencing methods have been previously described elsewhere [18]. Briefly, study participants collected faecal samples using sterile faecal collection tubes and samples were stored at 4 °C overnight until DNA extraction for a maximum duration of one day. Faecal samples were extracted using the PowerSoil DNA Extraction Kit (MO BIO Laboratories, Carlsbad, CA, USA) and subsequently cryopreserved at -80 °C until sequencing. Library preparation from whole faecal DNA was performed using the Nextera-XT® Illumina kit and sequenced in an Illumina Hi-Seq® platform.

### Sequence quality assessment and resistome identification

Quality filtering and human contamination removal from total sequences has been previously described elsewhere [18]. Shortly, sequence quality assessment and filtering were performed using FastQC and Trimmomatic software respectively and human contamination was eliminated using bwa software by aligning filtered reads to the human genome. Next, a mean of 31 million reads per individual were obtained.

Finally, ARIBA [49] (v2.14.6) pipeline was used with default settings to identify AMRD directly from filtered reads using CARD [50] (v3.1.4) as the reference database.

### Data processing and normalization

ARIBA resistome reports generated for each sample were filtered to remove variant information and were merged with CARD database in order to obtain CARD ontological information for the identified AMRD. Filtered resistome reports were unified in an AMRD abundance matrix containing the number of mapped reads to each AMRD for all samples. Mapped reads were normalized using the RPKM (Reads per Kilobase per Million mapped reads) method.

With the aim of achieving more interpretable results at functional level, normalized resistome abundances were grouped by AMR gene family CARD ontological category by aggregating RPKM values of AMRD belonging to each AMR gene families.

### Resistome compositional analysis

Resistome compositional analyses were performed from normalized AMRD matrix using R *vegan* v.2.6.2 package. Alpha diversity indexes were calculated using *diversity* function and differences in alpha diversity among groups were evaluated using a Wilcoxon rank sum test.

For beta diversity analysis, ecological distance matrix according to AMRD composition was calculated using Bray–Curtis dissimilarity index using *vegdist* function. Nonmetric multidimensional scaling (NMDS) approach was used for ordination using *metaMDS* function and visualized using *ggplot2* package. The PERMNOVA test was used to evaluate differences in beta diversity among groups using *adonis2* function. Resistome abundances grouped by AMR gene family were fitted onto NMDS ordination using *envfit* function and only AMR gene families significantly (*p* < 0.05) associated to an environmental vector of more than 0.2 NMDS length were represented. NMDS ellipses were drawn based on 0.95 confidence interval.

We performed PERMANOVA sequential and marginal multivariate analyses including significantly associated variables in the PERMANOVA univariate analysis. As this method does not accept missing values, we performed a first multivariate analysis including significant variables without missing values and a second multivariate analysis excluding missing values so all variables could be included in the analysis.

### Differentially abundance analysis

For this analysis, low-abundant AMRD (present in less than 10% of total samples) were filtered and grouped by AMR gene family CARD ontological category. Differentially abundant AMR gene families according to sexual preference, gene richness and microbiome enterotype were evaluated using a Wilcoxon rank sum test and the Benjamini–Hochberg correction was applied to correct multiple comparisons. For AMR gene families identified to be significantly different (adjusted *p* value < 0.05) in abundance between groups, median RPKM values per group were calculated. Significant AMR gene families with median RPKM values of zero in both comparison levels were excluded.

### Supplementary Information

Supplementary Material 1.

Supplementary Material 2.

Supplementary Material 3.

Supplementary Material 4.

Supplementary Material 5.

Supplementary Material 6.

Supplementary Material 7.

Supplementary Material 8.

## Data Availability

Raw sequences generated from Illumina HiSeq and study metadata are available in the National Center for Biotechnology Information (NCBI) repository (Bioproject accession number: PRJNA307231, SRA accession number: SRP068240).
