# Supplementary figures and images for: Gut resistome linked to sexual preference and HIV infection
Source: BMC Microbiol. 2024 Jun 8;24:201. doi: 10.1186/s12866-024-03335-z (PMC11162057; doi:10.1186/s12866-024-03335-z)

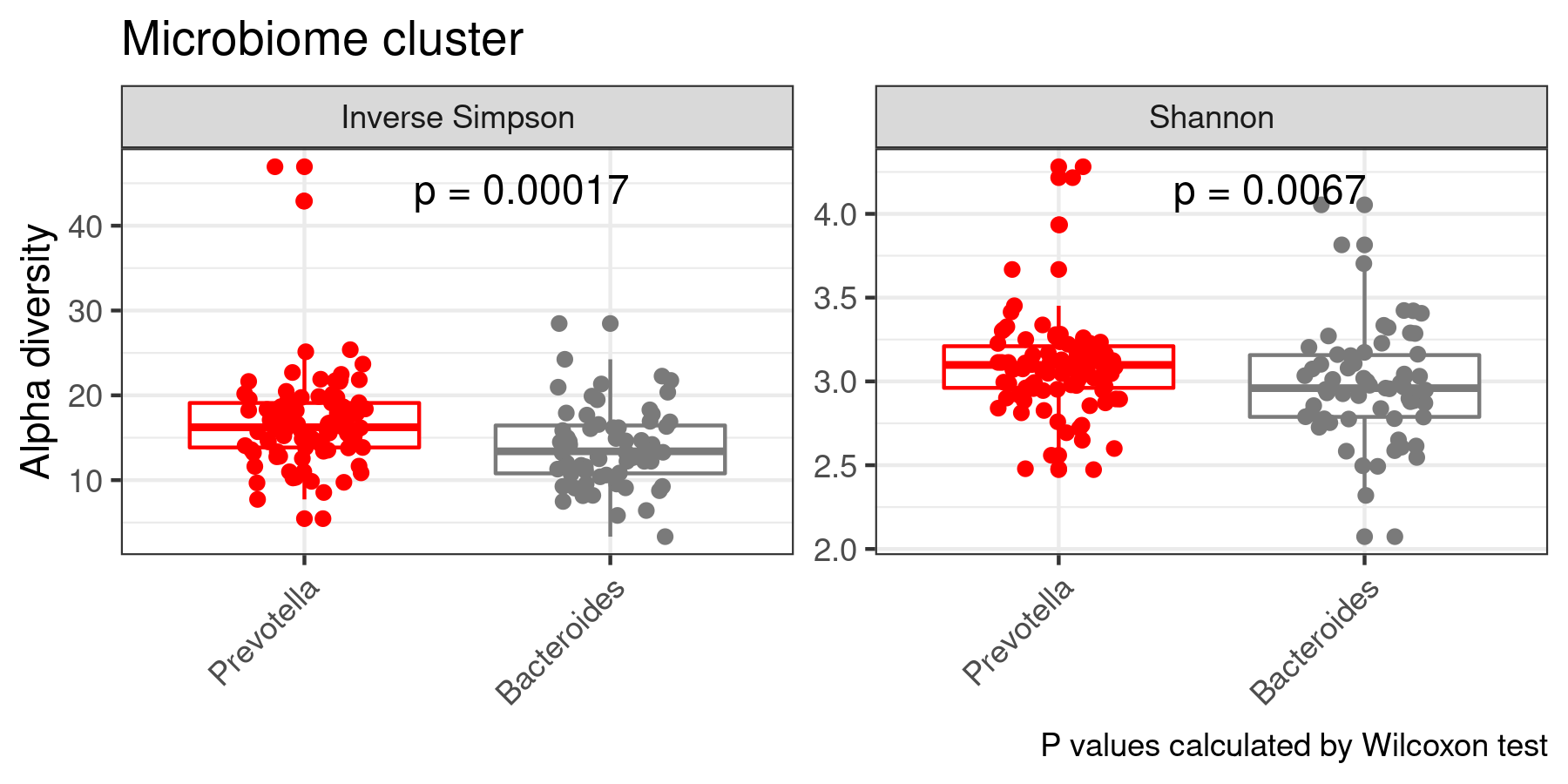

Supplement: Supplementary file 1 — Supplementary Material 1. [file 12866_2024_3335_MOESM1_ESM.tiff]

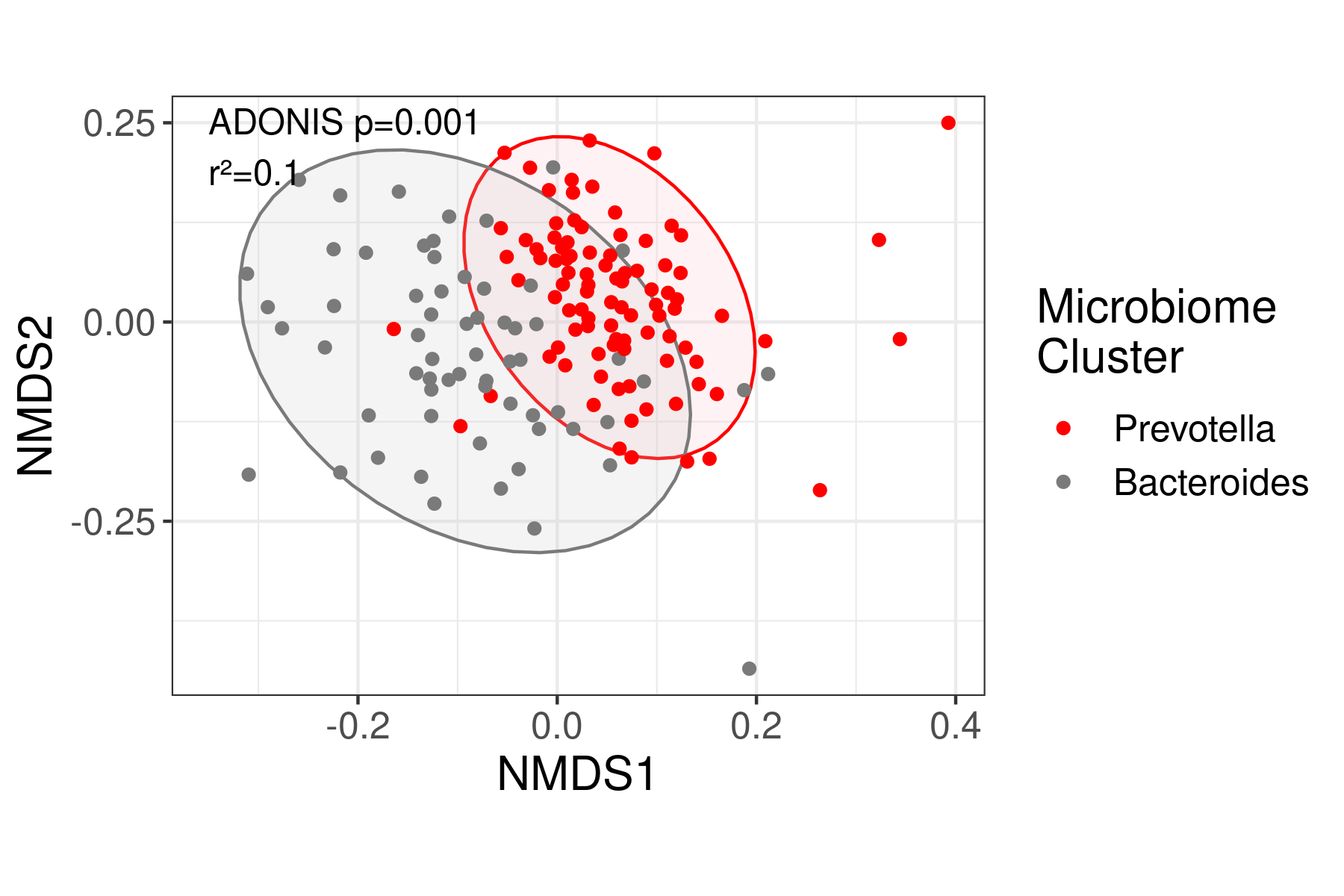

Supplement: Supplementary file 2 — Supplementary Material 2. [file 12866_2024_3335_MOESM2_ESM.tiff]

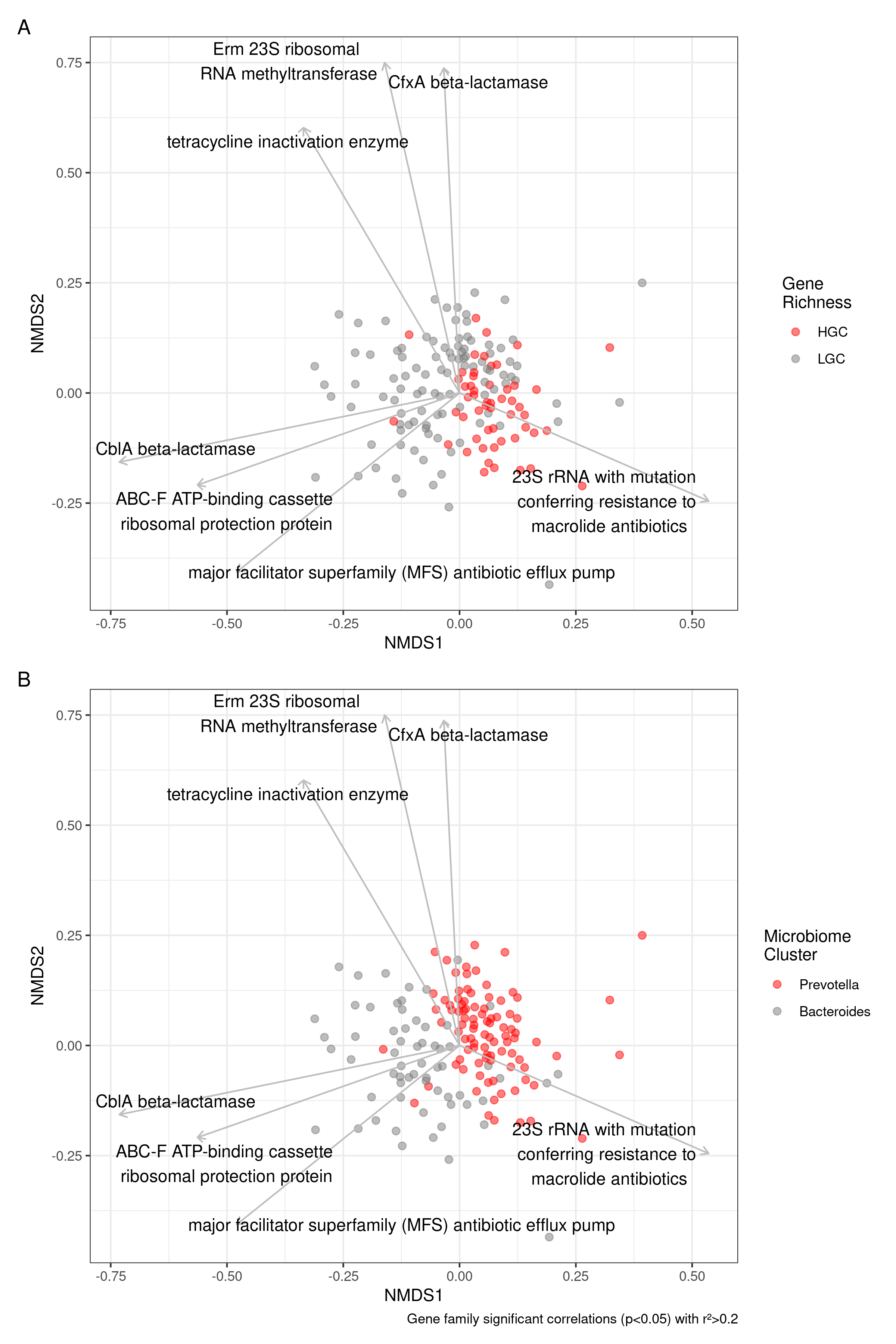

Supplement: Supplementary file 3 — Supplementary Material 3. [file 12866_2024_3335_MOESM3_ESM.tiff]
